# Neoplasm of uncertain behaviour of the breast—a retrospective study in a breast unit

**DOI:** 10.3332/ecancer.2018.839

**Published:** 2018-05-31

**Authors:** Xavier de Sousa, Pedro Santos Ferreira, Luís Branco, Jorge Simões, Matilde Gonçalves, Manuel Vítor Rigueira, Luís Cortez

**Affiliations:** 1 General Surgery Department, Setúbal Hospital Centre, São Bernardo Hospital, 2910-446 Setúbal, Portugal; 2 Breast Unit, General Surgery Department, Setúbal Hospital Centre, São Bernardo Hospital, 2910-446 Setúbal, Portugal; 3 Breast Unit, Obstetrics and Gynaecology Department, Setúbal Hospital Centre, São Bernardo Hospital, 2910-446 Setúbal, Portugal; 4 Pathology Department, Setúbal Hospital Centre, São Bernardo Hospital, 2910-446 Setúbal, Portugal

**Keywords:** neoplasm of uncertain behaviour of the breast, malignant potential, core needle biopsy, excisional biopsy

## Abstract

**Introduction:**

Breast lesions include a heterogeneous group of entities with variable clinical behaviour and morphological presentation, mostly classified as benign or malignant, with predictable behaviour. However, there are lesions with clinical, breast imaging and/or biopsy characteristics that do not clarify their nature. These lesions have an uncertain behaviour regarding their malignant potential at diagnosis.

We intend to relate the preoperative diagnosis of neoplasm of uncertain behaviour of the breast (NUnBB) regarding the core needle biopsy and the histological result after excisional biopsy.

**Methods:**

This is a retrospective study of patients submitted to local excision of breast lesions with a perioperative diagnosis of NUnBB, classified as 2383 at ‘International Statistical Classification of Diseases and Related Health Problems’ (ICD 9), between January 2007 and October 2016 in a breast unit.

**Results:**

Ninety-two cases with the diagnosis of NUnBB were analysed: 91 females with a mean age of 59 ± 14 years. All were submitted to local excision of breast lesion as ambulatory surgery with the following histology: 64% benign, 3% malignant potential and 33% malignant. Of those who presented malignant results, 69% underwent a surgical re-intervention for local control of the disease.

**Discussion:**

Regarding the considerable number of malignant lesions at final histology and the high percentage of which are re-operated, NUnBB should be treated with the same priority as a confirmed malignant neoplasm and whenever possible, using the most appropriate surgical technique.

## Introduction

Cells of the mammary gland present a high degree of phenotypic plasticity, which is reflected in the diversity of breast lesions, normally classified as benign or malignant [1]. However, some lesions include a heterogeneous group of entities, with a variable histology and clinical presentation [2] that does not clarify their nature at the moment of diagnosis. They consist of a broad spectrum of lesions with variable risk of progression, thus with an uncertain behaviour regarding their malignant potential [1, 3].

Borderline lesions with an uncertain behaviour, diagnosed with core needle biopsy (CNB) include atypical ductal hyperplasia (ADH), lobular neoplasia, papillary lesions (PL), complex sclerosing lesions (CSLs) and fibroepithelial lesions [3–13].

**ADH** is a lesion that exhibits a spectrum from mild atypical changes to low grade ductal carcinoma *in situ* (DCIS) [9, 12–14]. However, it does not have any recognisable symptoms or breast imaging abnormalities [6]. Usually, biopsies are done due to microcalcifications in mammograms [9, 15]. The amount of tissue available in a biopsy is insufficient for a definitive diagnosis. Up to 40% of ADH is upgraded to DCIS or invasive carcinoma [12, 13], having ADH means an increased risk of developing breast cancer in the future [9, 15]. Although it is not certain whether ADH is a marker of breast cancer risk or a precursor lesion, these lesions should be excised for definitive characterisation [9, 12, 13].

**CSLs**, which include radial scars, are characterised by dense stromal fibrosis with stellate proliferation that distorts the surrounding tissues. It may mimic the malignancy on radiographic exams. Malignancy may not be reliably excluded after biopsy and the lesion may be recommended for excision [9, 12–15].

**PLs** encompass a broad spectrum of benign, atypical and malignant neoplasms [16]. CNBs may present only with a sample of PLs, in which only a segment of benign epithelium may represent carcinoma elsewhere [12]. PLs are at a greater risk of developing breast cancer; therefore, excision is advised [13, 14].

**Fibroepithelial lesions** are a heterogeneous group of lesions mostly comprising fibroadenomas and phyllodes tumours [12, 15]. Both share some overlapping features; however, accurate differentiation between them may sometimes be difficult with CNB. Diagnosing phyllodes tumour with CNB may be equivocal, and when a cellular fibroepithelial lesion is difficult to classify, it should be totally excised for definitive classification [12, 13, 17].

The purpose of this study is to relate the preoperative diagnosis of neoplasm of uncertain behaviour of the breast (NUnBB) regarding the results of the CNB with the histological result after its excisional biopsy and how this would impact the patients’ lives.

## Methods and materials

A retrospective study was performed regarding all patients submitted to an excisional biopsy of a diagnosis of NUnBB (ICD 9 2838), between January 2007 and October 2016, in a breast unit. Hospital board approval was obtained and written informed consent of the patients was not required. All data were obtained consulting the patients’ hospital file. The variables considered were age and gender, result of CNB, histological result of excisional biopsy of the breast lesion and follow-up. Patients who did not present all this information were excluded.

All patients included had CNB showing a NUnBB, a discordant breast image result or a breast lesion with a suspicious clinical behaviour.

All CNB results were classified as benign, as breast lesions with indication for excision (BLIE) or as insufficient and were related with the histological result of the excised breast lesion. The excised breast lesions were classified as benign, as with malignant potential (WMP) or as malignant.

CNB classified as benign was fibroepithelial lesions and cysts, all with high breast imaging/clinical suspicion of malignancy; as BLIE was CSL/radial scars, PL and ADH most with breast imaging/clinical suspicion of malignancy; and as insufficient were breast lesions with breast imaging/clinical suspicion of malignancy but in which CNB was inconclusive.

Histological result of the excisional biopsy classified as benign were sclerosing lesions, intraductal papillomas, usual ductal hyperplasias, fibroadenomas and cysts; as WMP was ADH; and as malignant was *in situ* and invasive carcinomas.

Follow-up after excisional biopsy was in Senology consultation. According to the definitive histological result of malignant breast lesions, patient destination was oncology consultation or surgical re-intervention.

Statistical analysis was performed using the IBM SPSS programme, version 21. A descriptive analysis of the variables was performed. Categorical variables are presented as frequencies and percentages, and numeric variables, when shown to have normal distribution, are presented with mean and standard deviation. We tested the difference between the mean of the ages of the three groups (benign, WMP and malignant) using one-way ANOVA. Statistically significant results were shown with *p*-values < 0.05.

## Results

During the mentioned period, there were a total of 123 patients with the preoperative diagnosis of NUnBB. Thirty-one were excluded for not having all the information required for the study. A total of 92 patients were included in this study.

Of the 92 patients, 91 (99%) were female. The mean age was 59 ± 14 years with age range between 25 and 93 years (Table 1).

Breast imaging results demonstrated that 72% of all patients presented with breast imaging-reporting and data system (BI-RADS) higher than 3% and 11% did not have a BI-RADS classification on the report (Table 2). The breast images without report showed either highly suspicious breast nodes, distortion of the breast parenchyma or irregular microcalcifications.

CNB results were 5% benign, 83% BLIE and 12% insufficient (Table 3).

All patients were submitted to excisional biopsy of the breast lesion in ambulatory surgery.

The histological results of the excisional biopsy were: 64% benign, 3% WMP and 33% malignant (Table 3).

Of the patients with CNB showing BLIE, excisional biopsy of the breast lesion showed that 24 (32%) were malignant and 29 (64%) were benign (Table 4).

Of the patients with malignant breast lesions, CNB showed that 24 (80%) were BLIE and 5 (17%) were insufficient (Table 4).

In comparison with the result of benign breast lesion after excision, it has been demonstrated that there was a statistical significant age difference (p = 0,017) between the benign breast lesions after excision and the malignant breast lesions. The mean age for benign breast lesions was 56 ± 14 years and for malignant was 64 ± 13 years.

Of the breast lesions with malignant results, 21 (69%) had to be submitted for a surgical re-intervention (Table 5).

## Discussion

No clinical, imaging or biopsy characteristics have been shown to accurately identify breast lesions of uncertain behaviour [4, 5], thus not being able to identify which lesion will progress to malignancy or not [6], obliging surgeons to totally excise these lesions in order to obtain the final diagnosis.

The treatment strategy of breast lesions with uncertain malignant potential at diagnosis is not well established [4]. There is a risk of either unnecessary over or under treatment, although an accurate treatment is decisive to prevent invasive cancer [5–7].

The purpose of this study was to relate the preoperative diagnosis NUnBB regarding the results of the CNB with the histological result after its excisional biopsy and impact in the patients’ lives.

Breast lesions with CNB that show diagnostic doubt in relation to their nature need to be excised for an accurate and definite histological characterisation.

There are not many studies in the literature describing NUnBB and in what way it may impact the patient’s life. Although only a small number of patients were evaluated, one of the limitations of this study, the results showed that:

- 59 (64%) of the patients had a benign result after excisional biopsy. These results excluded the need for any following intervention besides adequate prophylactic breast imaging according to the patient’s age.
- 3 (3%) of the lesions excised had the result of WMP, which were ADH. Given these results, tight surveillance of these patients is crucial, because of the increased risk of developing breast cancer in the future [9, 15].
- 30 (33%) of the breast lesions excised with the diagnosis of NUnBB had a histological diagnosis of malignant neoplasm.

As is well known, breast malignancy affects older patients, with the increased risk of developing breast cancer above 60 years of age. This study showed that the patients with the result of breast malignancy belonged to group approximately a decade older than to the group whose excisional biopsy demonstrated benign breast lesions.

When facing the possibility of breast malignancy, its diagnosis should be made as soon as possible, as it may greatly impact a patient’s life. Breast lesions with the preoperative diagnosis of NUnBB in our breast unit were treated with ‘normal priority’, with a longer response time when compared to breast lesions diagnosed as malignant before treatment. Malignant breast neoplasms are treated within 30 days after diagnosis, while NUnBB was treated between 30 and 180 days, thus delaying the treatment of a possible malignant neoplasm.

All patients with malignant neoplasm were followed in oncology consultations, but 69% of them had to be re-operated. Sentinel lymph node biopsy was performed to all of the re-operated patients, as it is indicated in the early-stage invasive breast cancer [18]. Five patients had invading margins on excisional biopsy and had to have margin excision, one of them with the need for mastectomy.

## Conclusion

Although not many breast lesions are diagnosed as NUnBB, in this study, the third one was shown to be malignant after the excisional biopsy.

These results show the need for a faster response when facing a preoperative diagnosis of NUnBB, especially in older age groups.

Bearing this in mind, it may be important to treat these patients under the following terms: surgery in an adequate time, with ‘priority’ scheduled treatment; appropriate surgical technique, making sure all margins are tumour free so as to avoid a surgical re-intervention and marking the area with metal clips for eventual radiotherapy.

## Conflicts of interest

The authors declare that they have no conflicts of interest.

## Author’s contributions

Pedro Santos Ferreira has contributed equally to this article, and should also be considered first author.

## Figures and Tables

**Table 1.** Population, *N* = 92.

Gender (%)	
Male	1
Female	99
**Age (years)**	59 ± 14 [25–93]

**Table 2.** Breast imaging results, *N* = 92.

BI-RADS–*n* (%)	CNB—*n*	Excisional biopsy—*n*
2–4 (4)	Benign—1; BLIE—3	Benign—4
3–12 (13)	Benign—1; BLIE—11	Benign—11; Malignant potential—1
4–61 (66)	Benign—3; BLIE—49; Insufficient—9	Benign—39; Malignant potential—2; Malignant—20
5– 5 (5)	BLIE—4; Insufficient—1	Malignant—5
U– 10 (11)	BLIE—9; Insufficient—1	Benign—5; Malignant—5

BI-RADS—breast imaging-reporting and data system; CNB—core needle biopsy; BLIE—breast lesion with indication for excision;

U—unknown BI-RADS

CNB results were 5% benign, 83% BLIE and 12% insufficient.

**Table 3.** Histological results, *N* = 92.

CNB—*n* (%)	
Benign	5 (5)
BLIE	76 (83)
Insufficient	11 (12)
**Excisional biopsy—n (%)**	
Benign	59 (64)
WMP	3 (3)
Malignant	30 (33)

CNB—core needle biopsy; BLIE—breast lesion with indication for excision; WMP—with malignant potential

**Table 4.** Correlation of CNB with excisional biopsy, *N* = 92.

CNB (*n*)	Excisional biopsy (*n*)
Benign	
Fibroepithelial lesion (4)	Fibroadenoma (4)
Cyst (1)	Invasive papillary carcinoma (1)
BLIE	
ADH (19)	Benign (8); ADH (1); DCIS (7); DCIS with microinvasion (1); Invasive carcinoma (2)
CSL/radial scar (23)	Benign (18); DCIS (2); Invasive Carcinoma (3)
PL (34)	Benign (23); ADH (2); DCIS (2); Intracystic papillary carcinoma (1); Invasive papillary carcinoma (6)
Insufficient (12)	Benign (6); DCIS (1); Cribiform invasive carcinoma (1); Invasive carcinoma (3)

BLIE—breast lesion with indication for excision; DCIS—ductal carcinoma *in situ*; ADH—atypical ductal hyperplasia

**Table 5.** Destination following Senology consultation, *N* = 30.

Oncology consultation (n)	30
Surgical re-intervention (n)	
SNLB	16
Margin excision + SNLB	4
Mastectomy + SNLB	1

SNLB—sentinel lymph node biopsy
